# Retraction: Azuma, K. et al. Chitin, Chitosan, and Its Derivatives for Wound Healing: Old and New Materials. *J. Funct. Biomater*. 2015, *6*, 104–142

**DOI:** 10.3390/jfb9020038

**Published:** 2018-06-07

**Authors:** Kazuo Azuma, Ryotaro Izumi, Tomohiro Osaki, Shinsuke Ifuku, Minoru Morimoto, Hiroyuki Saimoto, Saburo Minami, Yoshiharu Okamoto, Journal of Functional Biomaterials Editorial Office

**Affiliations:** 1 Department of Veterinary Clinical Medicine, School of Veterinary Medicine, Tottori University, 4-101 Koyama-minami, Tottori 680-8553, Japan; tosaki@muses.tottori-u.ac.jp (T.O.); saburominami@ncn-t.net (S.M.); yokamoto@muses.tottori-u.ac.jp (Y.O.); 2 Graduate School of Engineering, Tottori University, 4-101 Koyama-minami, Tottori 680-8553, Japan; jastam0ment@gmail.com (R.I.); sifuku@chem.tottori-u.ac.jp (S.I.); saimoto@chem.tottori-u.ac.jp (H.S.); 3 Division of Instrumental Analysis, Research Center for Bioscience and Technology, Tottori University, 4-101 Koyama-minami, Tottori 680-8550, Japan; morimoro@chem.tottori-u.ac.jp; 4 MDPI, St. Alban-Anlage 66, 4052 Basel, Switzerland

The *Journal of Functional Biomaterials* Editorial Office have been made aware that some parts of the article [1] are duplicated from other publications [2,3,4,5].

MDPI is a member of the Committee on Publication Ethics and takes the responsibility to enforce strict ethical policies and standards very seriously. To ensure the addition of only high quality scientific works to the field of scholarly publication, Reference [1] is retracted and shall be marked accordingly. The article is retracted with the agreement of all authors. We apologize to the readership of *Journal of Functional Biomaterials* for any inconvenience caused.
